# Genetic Liability to Psychiatric Disorder and Risk of Musculoskeletal Diseases: A Bidirectional Mendelian Randomization Study

**DOI:** 10.62641/aep.2196

**Published:** 2026-10-15

**Authors:** Xiaohai Luo, Ning Wu, Zhaofu Wang, Jingzu Ma, Xiaoyu Zhang

**Affiliations:** ^1^Department of Orthopedics, People's Hospital of Ningxia Hui Autonomous Region, 750002 Yinchuan, Ningxia, China; ^2^Department of Orthopedics, Ningxia Integrated Hospital of Traditional Chinese and Western Medicine, 750021 Yinchuan, Ningxia, China; ^3^The First Clinical Medical College, Lanzhou University, 730000 Lanzhou, Gansu, China

**Keywords:** major depressive disorder, autism spectrum disorder, Mendelian randomization, genome-wide association study, osteoarthritis

## Abstract

**Background:**

Psychiatric disorders, particularly major depressive disorder (MDD), are increasingly recognized as systemic diseases that may predispose individuals to a range of physical comorbidities. Epidemiological studies have reported associations between depression and musculoskeletal diseases; however, causal inferences remain limited due to confounding factors and reverse causation. This study aims to investigate the bidirectional causal relationships between common psychiatric disorders and musculoskeletal outcomes using a Mendelian randomization (MR) framework.

**Methods:**

A bidirectional two-sample MR analysis was conducted using summary statistics from a large-scale genome-wide association study (GWAS) in European ancestry populations. Genetic instruments for seven psychiatric disorders were obtained from the Psychiatric Genomics Consortium, while musculoskeletal outcomes were sourced from the IEU Open GWAS and UK Biobank. Causal effects were primarily estimated using an inverse variance weighted model, with sensitivity analyses conducted using the weighted median (WM) and MR-Egger methods. Established diagnostic methods were employed to assess horizontal pleiotropy, heterogeneity, and robustness, and false discovery rate (FDR) corrections were applied to account for multiple testing.

**Results:**

The genetic liability to MDD showed the most consistent evidence of association with osteoarthritis (OA) and osteomyelitis (OM). The association with OA appeared to be the most stable overall, while the findings regarding OM should be interpreted more cautiously due to the relatively limited number of available instrumental variables. Nominal associations were also observed for fracture and osteoporosis (OP) in relation to MDD, as well as between autism spectrum disorder (ASD) and fracture risk and between anorexia nervosa (AN) and fracture risk; however, these findings were either modest in magnitude or did not remain significant after multiple-testing correction, and thus should be considered exploratory results. There was no strong evidence supporting a reverse causal effect of musculoskeletal diseases on psychiatric disorders.

**Conclusions:**

This MR study suggests that genetic liability to MDD may be associated with susceptibility to specific musculoskeletal diseases, particularly OA, and more cautiously, OM. These findings should be interpreted with caution, especially for associations based on limited instrumental support or modest effect sizes, and require replication in different populations.

## Introduction

Psychiatric disorders are one of the leading causes of disability worldwide and are increasingly recognized as diseases with systemic relevance that extend beyond mere mental health. Major depressive disorder (MDD), bipolar disorder (BD), and schizophrenia (SCZ) are persistently associated with adverse physical health outcomes, including metabolic, cardiovascular, and musculoskeletal diseases [1, 2, 3]. Among these conditions, musculoskeletal diseases such as osteoporosis (OP), fractures, and osteoarthritis (OA) significantly contribute to long-term disability and reduced quality of life, particularly in older populations. Recent genetic and epidemiological studies suggest that depression may be associated with an increased risk of OP, fractures, and OA [2, 3]. However, whether these associations reflect causal relationships remains uncertain, as traditional epidemiological studies are susceptible to residual confounding, medication effects, and reverse causation.

Mendelian randomization (MR) uses germline genetic variation as instrumental variables to enhance causal inference by reducing confounding and minimizing reverse causation [4, 5]. Previous MR and genomic studies have examined specific links between psychiatric disorders and musculoskeletal outcomes. For example, Tang *et al*. [6] reported no significant causal relationship between mental disorders and OP, while Barowsky *et al*. [7] identified a shared genetic basis between major depression and OA pain. However, these studies have mostly focused on isolated disorder–outcome pairs, such as mental disorders with OP or major depression with OA-related phenotypes, with less attention given to the broader spectrum of psychiatric disorders or less-studied musculoskeletal outcomes [2, 7]. As a result, it remains unclear whether the genetic liability to common psychiatric disorders is associated with multiple musculoskeletal diseases and whether musculoskeletal conditions, in turn, have an impact on psychiatric disorders. Addressing these gaps may help clarify the broader implications of psychiatric liability for physical health and provide a more integrated approach to mental and musculoskeletal health care.

To address these limitations, we conducted a bidirectional two-sample MR study to evaluate the relationship between common psychiatric disorders and musculoskeletal diseases. Specifically, we examined the genetic liability of seven major psychiatric disorders: MDD, BD, SCZ, attention-deficit/hyperactivity disorder (ADHD), autism spectrum disorder (ASD), obsessive–compulsive disorder (OCD), and anorexia nervosa (AN) in relation to the risk of OP, fractures, OA, and osteomyelitis (OM). At the same time, we assessed the reverse direction to determine whether the genetic susceptibility of musculoskeletal diseases influences psychiatric disorders. By utilizing summary data from large-scale genome-wide association study (GWAS) in European ancestry populations and applying complementary MR estimation methods with multiple sensitivity analyses, this study aims to clarify the directionality and relative robustness of these associations, with a particular focus on a broader multiple exposure framework, bidirectional design, and the inclusion of OM as a less-studied musculoskeletal outcome.

## Materials and Methods

### Study Design

We applied a bidirectional two-sample MR framework to assess the relationship between seven psychiatric disorders (MDD, BD, SCZ, ADHD, ASD, OCD, and AN) and four musculoskeletal outcomes (OP, fractures, OA, and OM). The analysis followed three MR assumptions: (i) the IVs are strongly associated with the exposure; (ii) the IVs are independent of confounders; and (iii) the IVs affect the outcome only through the exposure pathway [8, 9]. The report adhered to the STROBE-MR recommendations (covering items related to design, instrument selection, harmonization, and sensitivity analyses) (Fig. 1) [10].

**Fig. 1.**
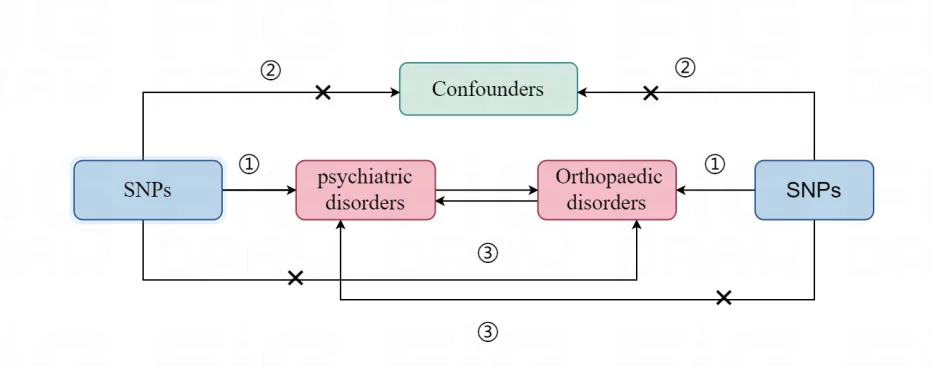
**Study workflow for the bidirectional two-sample MR**. SNPs, single-nucleotide polymorphisms; MR, Mendelian randomization.

### Data Sources

The summary-level GWAS data for psychiatric traits were obtained from the PGC; the orthopaedic outcomes were sourced from the IEU Open GWAS and UKB resources. All datasets were restricted to participants of European ancestry to reduce population stratification. The access numbers, phenotype definitions, and sample sizes of the datasets are listed in Table 1; the sample size for psychiatric traits ranged from N = 33,925 to 500,199, while the sample size for orthopaedic outcomes ranged from N = 407,746 to 484,598 [11]. The assessment of sample overlap was based on a conservative evaluation of the alliance documents, which is expected to be limited; although individual-level overlap information for all contributing datasets is not available, GWAS for exposures and outcomes were extracted from different large-scale resources whenever possible. If there is residual sample overlap, it may bias the estimates towards observational associations, particularly when the instrumental variables are weak, and thus is considered a potential limitation.

**Table 1.** **GWAS data sources and sample characteristics for psychiatric disorders and musculoskeletal outcomes**.

Exposure or outcome	ID	Year	Sample size	Consortium	Ethnicity
MDD	ieu-b-102	2019	500,199	PGC	European
BD	ieu-b-5110	2021	413,466	PGC	European
SCZ	ieu-b-5102	2022	127,906	PGC	European
ADHD	ieu-a-1183	2017	55,374	PGC	European
ASD	ieu-a-1185	2017	46,351	PGC	European
OCD	ieu-a-1189	2017	33,925	PGC	European
AN	ieu-a-1186	2017	14,477	PGC	European
OP	ebi-a-GCST90038656	2021	484,598	NA	European
Fracture	ebi-a-GCST90013913	2021	407,746	NA	European
OA	ebi-a-GCST90038686	2021	484,598	NA	European
OM	ieu-b-4975	2021	486,484	UK Biobank	European

Abbreviations: GWAS, genome-wide association study; MDD, major depressive disorder; BD, bipolar disorder; SCZ, schizophrenia; ADHD, attention-deficit/hyperactivity disorder; ASD, autism spectrum disorder; OCD, obsessive–compulsive disorder; AN, anorexia nervosa; PGC, Psychiatric Genomics Consortium; NA, not available; OP, osteoporosis; OA, osteoarthritis; OM, osteomyelitis. Note: ID refers to the accession number of the corresponding GWAS dataset. Sample size refers to the total number of participants included in the original GWAS summary statistics.

### Instrument Selection

For each exposure, independent SNPs that are significantly associated under the conventional genome-wide significance threshold (*p* < 5 × 10^–⁢8^) should be selected as IVs whenever possible. When instrumental variables are scarce, a defined relaxed threshold (*p* < 5 × 10^–⁢6^) is applied to retain sufficient variants for exploratory analysis; these analyses should be interpreted with caution and marked in the Results and Supplementary materials. LD clustering is performed within a 10,000 kb window (European reference) at r^2^ < 0.001 to ensure independence. Variants with MAF < 0.01 or poor imputation quality are excluded. Instrument strength is summarized by the mean and minimum F-statistics; *F* > 10 is considered sufficient to mitigate weak-instrument bias [12, 13, 14]. The count and F-statistics for each pair of instruments are provided in **Supplementary Table 1**.

### Harmonization and Quality Control

The exposure and outcome datasets are harmonized to align effect alleles and effect directions. Palindromic SNPs with intermediate allele frequencies are excluded to minimize strand ambiguity. When there is no index SNP in the outcome dataset, proxy variants in high linkage disequilibrium (r^2^ > 0.8, European reference) are used if available. Steiger directional filtering is applied to retain variants that explain more variance in the exposure than in the outcome, thereby reducing the likelihood of reverse classification errors; variants that do not meet this criterion are excluded.

### Statistical Analysis

The primary estimate is obtained using the inverse-variance weighted (IVW) method under a random effects model, supplemented by sensitivity analyses using MR-Egger and weighted median (WM) estimators [15, 16, 17]. The IVW method is considered the primary estimate under the assumption that the included variants are valid instruments or that any level of pleiotropy is balanced on average, while WM and MR-Egger analyses are used to assess robustness under alternative assumptions. Cochran’s Q is used to assess heterogeneity among SNPs (*p* < 0.05 indicates heterogeneity), with random effects IVW estimates reported by default. The MR-Egger intercept test assesses the level of directional pleiotropy (*p* < 0.05 suggests the presence of pleiotropy). Outlier diagnostics are performed using MR-PRESSO global and distortion tests, as well as residual-based checks [18]; effects are examined through leave-one-out analyses. Effect sizes are reported as odds ratios (ORs) and 95% confidence intervals (CIs) for genetically predicted exposures per standard deviation increase.

### Multiple Testing and Robustness

Considering multiple exposures, outcomes, and bidirectional tests, the Benjamini–Hochberg false discovery rate (FDR) procedure (α = 0.05) was used to address multiplicity. Both raw *p* and FDR-adjusted *p* (*p*_adj) are reported. Results are considered more robust when they remain significant after FDR correction and show approximately consistent direction in complementary MR estimators, while nominal results (*p* < 0.05 but *p*_adj ≥ 0.05) are interpreted as exploratory. For readability, only selected nominally significant or other clinically relevant exposure-outcome pairs are presented in the main text table, without displaying individual non-significant results.

### Software and Reproducibility

The analysis was conducted using R version 4.3.1 (R Foundation for Statistical Computing, Vienna, Austria) and RStudio (Posit Software, PBC, Boston, MA, USA), with the TwoSampleMR v0.5.7 and MR-PRESSO v1.0 R packages, employing standard libraries for data processing and visualization. Reproducible materials, namely, R scripts, package versions, session information, IV lists, and harmonization logs, are provided in the Supplementary Materials and comply with the data usage policies summarized alongside Table 1.

## Results

### Instrument Selection and Strength

After performing LD clustering and quality control, independent SNPs related to psychiatric disorders and orthopaedic outcomes were retained as IVs (**Supplementary Table 1**). No significant linkage disequilibrium was observed between the selected instrumental variables. All selected variants showed strong associations with their respective exposures, with mean and minimum F-statistics exceeding 20, indicating sufficient instrument strength. The harmonized datasets were free from strand ambiguity, and the Steiger filter confirmed the correct causal direction.

### Causal Effects of Psychiatric Disorders on Orthopaedic Diseases

For ease of reading, Table 2 presents selected major MR estimates, focusing on nominally significant or other relevant associations. Using the IVW model as the primary estimator, the genetic liability to MDD shows the most consistent evidence of association with OA (OR = 1.010, 95% CI 1.006–1.014, *p* < 0.001) and OM (OR = 1.457, 95% CI 1.258–1.687, *p* = 0.001), while additional IVW associations with fractures (OR = 1.092, 95% CI 1.024–1.165, *p* = 0.008) and OP (OR = 1.002, 95% CI 1.000–1.004, *p* = 0.014) were also observed. Both the WM and MR-Egger estimators produced consistent directional results for the MDD–OA and MDD–OM associations, supporting these findings as major signals in the analysis (Table 2, Figs. 2, 3). However, the association between MDD and OM should be interpreted with caution, as it is based on a relatively limited number of instrumental variants (Table 2, Fig. 3). The observed association between MDD and OP is also modest in magnitude, so its clinical or biological relevance should not be overstated (Table 2, Fig. 2).

**Table 2.** **Selected primary MR estimates from psychiatric disorders to musculoskeletal outcomes**.

Exposure–Outcome	Method	β	SE	OR	95% CI	*p*-value
MDD–Fracture	MR Egger	0.045	0.134	1.046	0.804–1.360	0.740
	WM	0.052	0.040	1.054	0.976–1.138	0.195
	IVW	0.088	0.033	1.092	1.024–1.165	0.008
MDD–OP	MR Egger	0.001	0.004	1.001	0.993–1.009	0.886
	WM	0.003	0.001	1.003	1.000–1.005	0.039
	IVW	0.002	0.001	1.002	1.000–1.004	0.014
MDD–OA	MR Egger	0.006	0.008	1.006	0.991–1.021	0.440
	WM	0.009	0.003	1.009	1.004–1.015	0.001
	IVW	0.010	0.002	1.010	1.006–1.014	<0.001
MDD–OM	MR Egger	0.097	0.304	1.006	0.991–1.021	0.750
	WM	0.337	0.105	1.009	1.004–1.015	0.001
	IVW	0.376	0.075	1.457	1.258–1.687	0.001
ADHD–Fracture	MR Egger	0.119	0.064	0.888	0.783–1.007	0.070
	WM	0.031	0.023	1.032	0.988–1.077	0.161
	IVW	0.036	0.016	1.036	1.004–1.070	0.028
ASD–Fracture	MR Egger	0.067	0.055	1.070	0.959–1.192	0.234
	WM	0.011	0.027	1.011	0.961–1.065	0.665
	IVW	0.041	0.020	1.042	1.002–1.082	0.039
AN–Fracture	MR Egger	0.017	0.046	0.983	0.983–1.076	0.717
	WM	0.038	0.020	0.963	0.925–1.002	0.062
	IVW	0.041	0.017	0.960	0.928–0.993	0.018
Fracture–MDD	MR Egger	0.011	0.065	0.989	0.870–1.124	0.865
	WM	0.048	0.026	1.050	0.997–1.104	0.063
	IVW	0.064	0.020	1.066	1.024–1.110	0.002

Abbreviations: MDD, major depressive disorder; ADHD, attention-deficit/hyperactivity disorder; ASD, autism spectrum disorder; AN, anorexia nervosa; OP, osteoporosis; OA, osteoarthritis; OM, osteomyelitis; MR, Mendelian randomization; WM, weighted median; IVW, inverse-variance weighted; OR, odds ratios; CI, confidence interval; SE, standard error.

**Fig. 2.**
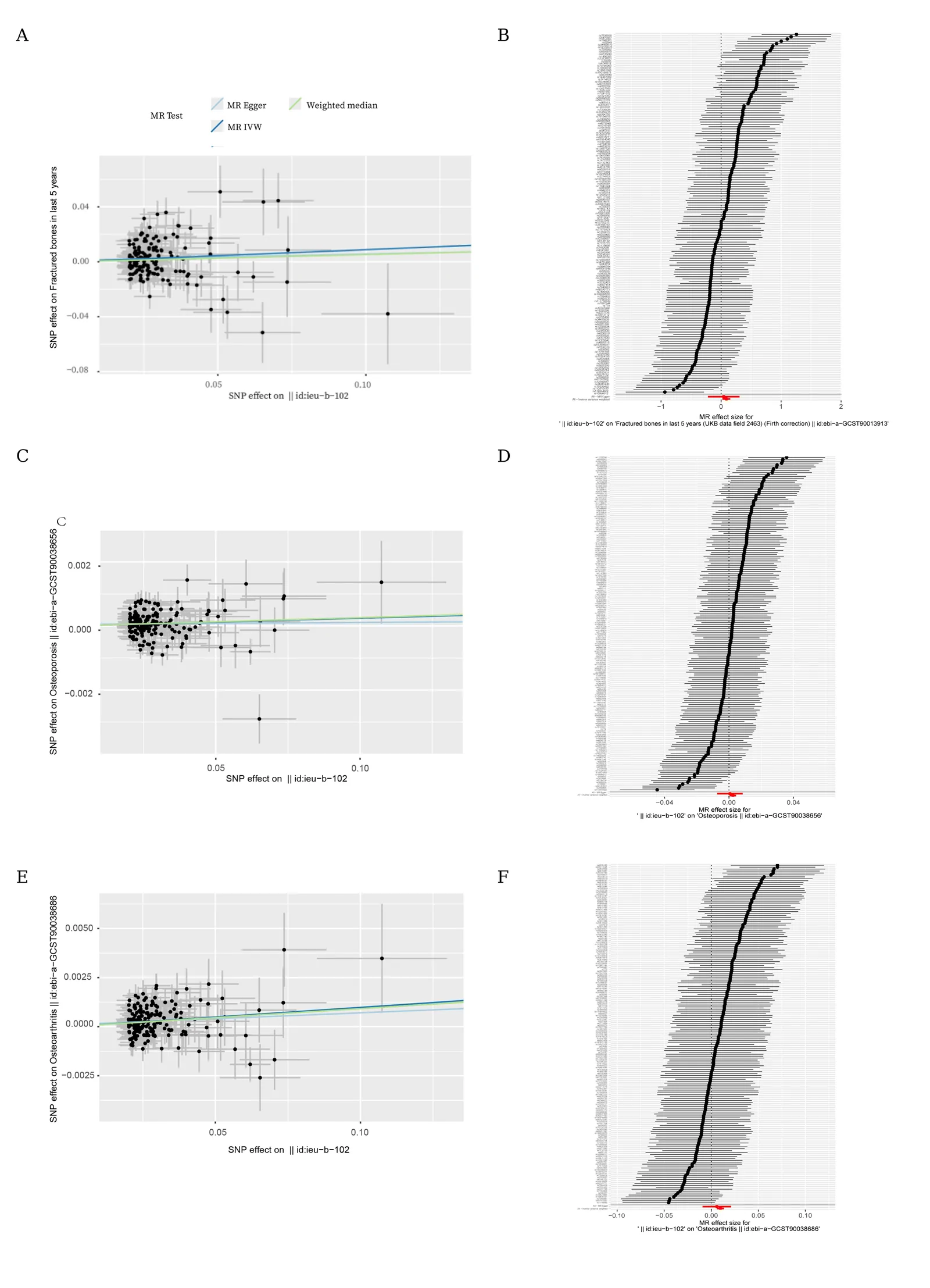
**Scatter plots and plots of leave-one-out for selected MR analyses of MDD and musculoskeletal outcomes**. (A) Scatter plot for MDD and fracture. (B) Forest plot for MDD and fracture. (C) Scatter plot for MDD and OP. (D) Forest plot for MDD and OP. (E) Scatter plot for MDD and OA. (F) Forest plot for MDD and OA. In the scatter plots, the x-axis is labelled “Exposure variable”, and the y-axis is labelled “Outcome variable”. Lines represent IVW, WM, and MR-Egger estimates. In the leave-one-out plots, each point represents the IVW estimate obtained after sequential omission of one SNP. Abbreviations: MR, Mendelian randomization; MDD, major depressive disorder; OP, osteoporosis; OA, osteoarthritis; IVW, inverse-variance weighted; WM, weighted median; SNP, single-nucleotide polymorphism.

**Fig. 3.**
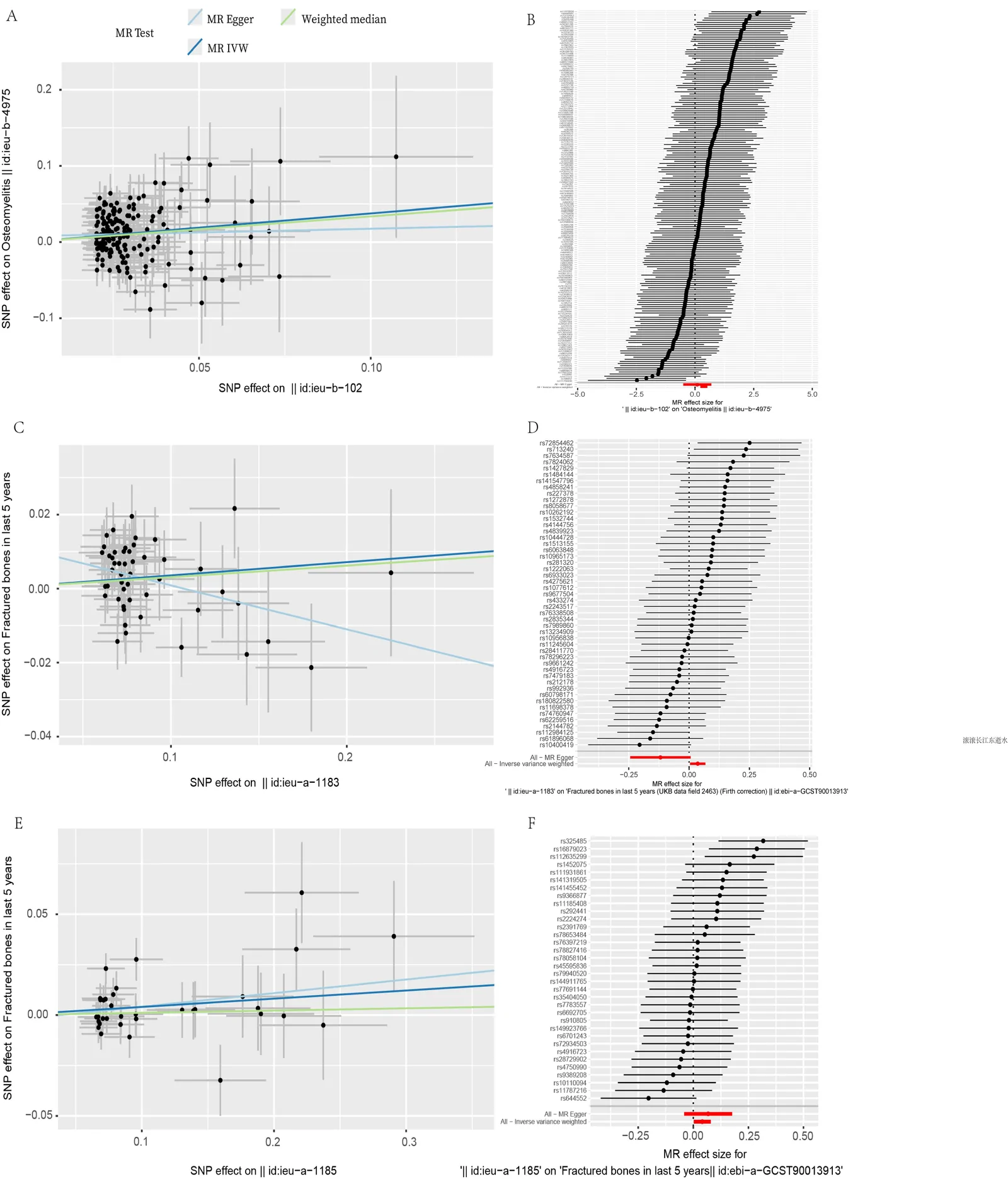
**Scatter plots and plots of leave-one-out for selected MR analyses**. (A) Scatter plot for MDD and OM. (B) Forest plot for MDD and OM. (C) Scatter plot for ADHD and fracture. (D) Forest plot for ADHD and fracture. (E) Scatter plot for ASD and fracture. (F) Forest plot for ASD and fracture. In the scatter plots, points represent SNP-specific effects, and lines represent IVW, WM, and MR-Egger estimates. In the leave-one-out plots, each point represents the IVW estimate obtained after sequential omission of one SNP. Abbreviations: MR, Mendelian randomization; MDD, major depressive disorder; ADHD, attention-deficit/hyperactivity disorder; ASD, autism spectrum disorder; OM, osteomyelitis; IVW, inverse-variance weighted; WM, weighted median; SNP, single-nucleotide polymorphism.

In addition, ADHD shows a nominal positive correlation with fractures (OR = 1.036, 95% CI 1.004–1.070, *P* = 0.028), and ASD also shows a similar nominal correlation (OR = 1.042, 95% CI 1.002–1.082, *p* = 0.039). In contrast, AN presents a nominal negative correlation with fracture risk (OR = 0.960, 95% CI 0.928–0.993, *p* = 0.018). However, these relationships did not maintain significance after FDR correction, and for the relationship between ADHD and fractures, the three estimation methods (IVW, MR-Egger, and WM) yielded inconsistent directions, suggesting that these signals should be considered exploratory rather than causal. No evidence was found for the causal effects of BD or SCZ on any orthopaedic outcomes (Table 2, Figs. 2, 3, 4).

**Fig. 4.**
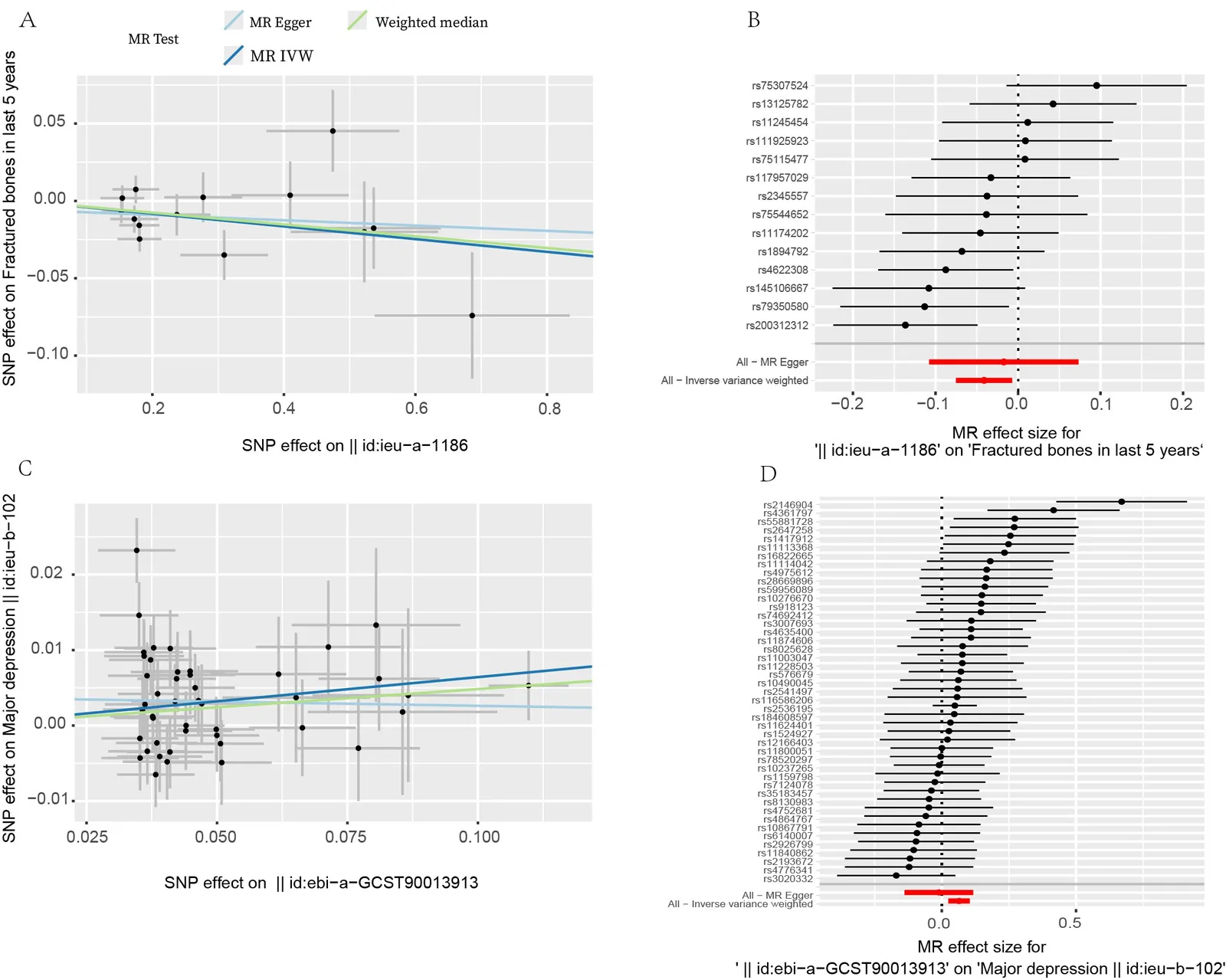
**Scatter plots and plots of leave-one-out for selected MR analyses**. (A) AN–fracture scatter plot. (B) Forest plot for AN–fracture. (C) Fracture–MDD scatter plot. (D) Forest plot for Fracture–MDD. In the scatter plots, lines indicate IVW, WM, and MR-Egger estimates. In the leave-one-out plots, each point indicates the IVW estimate after sequential removal of one SNP. Abbreviations: MR, Mendelian randomization; AN, anorexia nervosa; MDD, major depressive disorder; IVW, inverse-variance weighted; WM, weighted median; SNP, single-nucleotide polymorphism.

### Reverse Causal Effects of Orthopaedic Diseases on Psychiatric Disorders

In reverse-direction analyses, the IVW model indicated a nominal association between genetic liability and fractures, and increased the risk of MDD (OR = 1.066, 95% CI 1.024–1.110, *p* = 0.002). However, after considering the consistency of multiple testing and complementary MR methods, this signal did not meet the predefined robust inference criteria and should be interpreted with caution. In contrast, no robust reverse causal effects were observed from OP, OA, or OM to any psychiatric disorder. All reverse-direction estimates are summarized in Table 2 and illustrated in Fig. 4.

### Heterogeneity, Pleiotropy, and Sensitivity Analyses

The heterogeneity and horizontal pleiotropy test results for the selected MR analyses are summarized in Table 3. Cochran’s Q test found significant heterogeneity in the MDD–fracture analysis (MR-Egger: Q = 252, Q_df = 154, *p* < 0.001; IVW: Q = 252, Q_df = 155, *p* < 0.001) and in the MDD–OP analysis (MR-Egger: Q = 244, Q_df = 174, *p* < 0.001; IVW: Q = 245, Q_df = 175, *p* < 0.001). In contrast, no significant heterogeneity was detected in the MDD–OA, MDD–OM, ADHD–fracture, ASD–fracture, or AN–fracture associations. For the ADHD–fracture analysis, the MR-Egger intercept was statistically significant (intercept = 0.013, SE = 0.005, *p* = 0.016), suggesting a potential horizontal pleiotropy, while no clear directional pleiotropy was observed for other reported associations. To further assess the potential impact of pleiotropy, we applied the MR-PRESSO global and distortion tests; both yielded non-significant results, indicating that pleiotropy is unlikely to materially bias the primary causal estimates.

**Table 3.** **Heterogeneity and horizontal pleiotropy tests for selected MR analyses**.

Exposure	Outcome	Heterogeneity test				Horizontal pleiotropy test			
		Method	Q	Q_df	P	Method	Intercept	SE	P
MDD	Fracture	MR Egger	252	154	<0.001	MR Egger	0.001	0.004	0.738
		IVW	252	155	<0.001				
MDD	OP	MR Egger	244	174	<0.001	MR Egger	<0.001	<0.001	0.635
		IVW	245	175	<0.001				
MDD	OA	MR Egger	193	174	0.148	MR Egger	<0.001	<0.001	0.610
		IVW	194	175	0.157				
MDD	OM	MR Egger	200	174	0.083	MR Egger	0.008	0.008	0.344
		IVW	202	175	0.083				
ADHD	Fracture	MR Egger	49.2	47	0.384	MR Egger	0.013	0.005	0.016
		IVW	55.7	48	0.207				
ASD	Fracture	MR Egger	38	32	0.215	MR Egger	–0.003	0.005	0.611
		IVW	38.3	33	0.241				
AN	Fracture	MR Egger	20	12	0.067	MR Egger	–0.006	0.010	0.583
		IVW	20.5	13	0.083				
Fracture	MDD	MR Egger	71	43	0.005	MR Egger	0.004	0.003	0.232
		IVW	73.5	44	0.003				

MDD, major depressive disorder; ADHD, attention-deficit/hyperactivity disorder; ASD, autism spectrum disorder; AN, anorexia nervosa; OP, osteoporosis; OA, osteoarthritis; OM, osteomyelitis; MR, Mendelian randomization; IVW, inverse-variance weighted; SE, standard error.

In the reverse MR analyses, modest heterogeneity was detected for the fracture–MDD, but the MR-Egger intercept was not significant, and there was no clear evidence of directional pleiotropy. The funnel plot was broadly symmetrical, and leave-one-out sensitivity analyses suggested that no single SNP had a disproportionate effect on the estimates (**Supplementary Figs. 1,2,3**).

## Discussion

This bidirectional two-sample MR analysis suggests that genetic liability is associated with MDD and is related to an increased risk of OA and OM. Among the exposure–outcome pairs tested, the association with OA appears to be the most stable, as it remains significant after FDR correction and shows directionally consistent results in complementary estimators. The findings for OM are also statistically supported and directionally consistent, but should be interpreted with more caution due to the analysis relying on relatively limited instrumental variants. In contrast, associations involving fractures, OP, ASD, and AN are either modest in magnitude or did not remain significant after multiple testing correction, and should therefore be considered exploratory rather than definitive. The reverse-direction analyses did not yield robust evidence after FDR correction, suggesting that musculoskeletal diseases do not causally influence psychiatric disorders. Overall, these results support that the relationship between depressive liability and selected musculoskeletal outcomes is closer than the reverse relationship, while also highlighting the complexity of the connections between mental health and musculoskeletal health.

Our research findings are generally consistent with the findings of observational studies, which reported a higher incidence of fractures, lower bone density, and related musculoskeletal disorders in individuals with depression or affective symptoms [19, 20]. This analysis provides genetic epidemiological support for the association between liability to MDD and specific musculoskeletal outcomes. These differences may reflect variations in GWAS sources, instrument selection, statistical power, and harmonization strategies. Recent population-based evidence also suggests that depressive symptoms may be associated with the risk of incident fractures, particularly the risk of hip fractures in women, which is broadly consistent with our findings on liability levels [21].

For ASD and ADHD, early cohort studies reported an increased risk of fractures, whereas our estimates did not remain significant after FDR correction; thus, this should be considered exploratory research, suggesting that confounding factors or limited precision may partially explain the previous associations. Although prior literature has linked AN to skeletal fragility [6], the nominal negative correlation observed in our analysis is more likely attributed to instrument limitations, limited statistical power, or phenotype heterogeneity, rather than a true protective effect. In addition, we found no evidence supporting a causal effect of BD or SCZ on orthopaedic outcomes, which contrasts with observational reports of low bone mass and increased risk of hip fractures in SCZ [22, 23].

Several biological pathways may help explain the observed associations. Chronic low-grade inflammation, alterations in immune signaling, and dysregulation of the hypothalamic–pituitary–adrenal axis in MDD may reasonably affect cartilage homeostasis, bone remodeling, and host defense, thereby increasing susceptibility to OA and OM [24, 25]. The use of antidepressants, particularly selective serotonin reuptake inhibitors, has also been associated with changes in bone metabolism and an increased risk of fractures in observational studies, suggesting a potential treatment-related contribution [26]. Although drug use cannot be directly inferred from our MR design, it may represent a downstream or mediating pathway that contributes to a broader relationship between depressive liability and musculoskeletal vulnerability, rather than a simple direct causal chain. In contrast, the nominal reverse signal from fractures to MDD may reflect psychosocial stress, post-injury neuroinflammatory responses, or residual complexities, rather than a strong reverse causal relationship [27]. In terms of methodology, two-sample MR strengthens causal inference by clarifying directionality in comorbidity studies. In this study, multiple sensitivity analyses, including IVW, MR-Egger, WM, and MR-PRESSO, provided universally supportive evidence for the main associations, although the selected findings remain limited by the availability of instruments and should be interpreted with caution.

If these findings are replicated in future research, they may support a more integrated perspective on care, in which individuals with a higher tendency for depression are considered in the context of bone and joint health, along with broader consultations regarding physical activity, nutrition, and infection risk. Conversely, orthopaedic patients, particularly those who experience prolonged immobilization, may benefit from screening for depressive symptoms to optimize rehabilitation outcomes.

Several limitations should be acknowledged. First, MR relies on assumptions of relevance, independence, and exclusion restrictions, which cannot be fully verified and may be affected by unrecognized pleiotropy. Second, all summary statistics from GWAS are derived from populations of European ancestry, which limits the generalizability of these findings and underscores the need for replication in more diverse populations. Third, despite the application of MR-Egger and MR-PRESSO, their ability to detect subtle pleiotropy is limited, and residual bias cannot be completely ruled out. Fourth, the relatively small number of genome-wide significant instruments for certain exposures, as well as the selection for some outcome analyses such as OM, may reduce precision and estimate stability. In addition, when genome-wide significant variants are sparse, some exposure–outcome pairs were analyzed using relaxed SNP selection thresholds, which may have increased instrument availability at the cost of specificity and robustness. Finally, despite rigorous coordination, heterogeneity in phenotype definitions and imputation panels across different datasets may introduce variation; the overall findings, particularly exploratory associations, should be interpreted with caution.

## Conclusions

In conclusion, this bidirectional MR study suggests that genetic liability to MDD may increase susceptibility to certain musculoskeletal diseases, particularly OA and, under more cautious circumstances, OM. There is no strong evidence supporting a reverse causal effect of musculoskeletal diseases on psychiatric disorders. Given the limited number of instruments for the selected analyses, the modest magnitude of some associations, and the possibility that treatment-related or other mediating pathways may partially explain the observed relationships, these findings should be interpreted with caution. Further research in independent datasets and more diverse populations is needed to confirm these findings.

## Availability of Data and Materials

The data analyzed in this study are available from the corresponding author upon reasonable request.
