# Supplementary material for: Genetic Liability to Psychiatric Disorder and Risk of Musculoskeletal Diseases: A Bidirectional Mendelian Randomization Study
Source: Actas Esp Psiquiatr. 2026 Oct 15;54(5):1666–76. doi: 10.62641/aep.2196 (PMC13714466; doi:10.62641/aep.2196)
Supplement: Supplementary file 1 [file ActEsp-54-5-1666-1676-s1.zip › AEP-2196-Supplementary materials.pdf]

Supplementary table 1. Instrument counts and F-statistics.

Exposure	Outcome	Final inclusion of SNPs	Ethnicity	*F* value
Osteoporosis	MDD	44	European	>20
Osteoporosis	BIP	41	European	>20
Osteoporosis	Schizophrenia	46	European	>20
Osteoporosis	ADHD	36	European	>20
Osteoporosis	ASD	41	European	>20
Osteoporosis	OCD	43	European	>20
Osteoporosis	AN	44	European	>20
Fracture	MDD	45	European	>20
Fracture	BIP	47	European	>20
Fracture	Schizophrenia	47	European	>20
Fracture	ADHD	44	European	>20
Fracture	ASD	48	European	>20
Fracture	OCD	49	European	>20
Fracture	AN	50	European	>20
Osteoarthritis	MDD	43	European	>20
Osteoarthritis	BIP	35	European	>20
Osteoarthritis	Schizophrenia	46	European	>20
Osteoarthritis	ADHD	33	European	>20
Osteoarthritis	ASD	37	European	>20
Osteoarthritis	OCD	36	European	>20
Osteoarthritis	AN	37	European	>20
Osteomyelitis	MDD	10	European	>20
Osteomyelitis	BIP	9	European	>20
Osteomyelitis	Schizophrenia	9	European	>20
Osteomyelitis	ADHD	7	European	>20
Osteomyelitis	ASD	9	European	>20
Osteomyelitis	OCD	9	European	>20
Osteomyelitis	AN	10	European	>20

MDD	Osteoporosis	176	European	>20
MDD	Fracture	156	European	>20
MDD	Osteoarthritis	176	European	>20
MDD	Osteomyelitis	176	European	>20
BIP	Osteoporosis	165	European	>20
BIP	Fracture	165	European	>20
BIP	Osteoarthritis	165	European	>20
BIP	Osteomyelitis	165	European	>20
Schizophrenia	Osteoporosis	341	European	>20
Schizophrenia	Fracture	341	European	>20
Schizophrenia	Osteoarthritis	341	European	>20
Schizophrenia	Osteomyelitis	341	European	>20
ADHD	Osteoporosis	49	European	>20
ADHD	Fracture	49	European	>20
ADHD	Osteoarthritis	49	European	>20
ADHD	Osteomyelitis	49	European	>20
ASD	Osteoporosis	34	European	>20
ASD	Fracture	34	European	>20
ASD	Osteoarthritis	34	European	>20
ASD	Osteomyelitis	34	European	>20
OCD	Osteoporosis	11	European	>20
OCD	Fracture	11	European	>20
OCD	Osteoarthritis	11	European	>20
OCD	Osteomyelitis	11	European	>20
AN	Osteoporosis	14	European	>20
AN	Fracture	14	European	>20
AN	Osteoarthritis	14	European	>20
AN	Osteomyelitis	14	European	>20

Abbreviations: SNPs, single-nucleotide polymorphisms; MDD, major depressive disorder; BIP, bipolar disorder; ADHD, attention-deficit/hyperactivity disorder; ASD, autism spectrum

disorder; OCD, obsessive–compulsive disorder; AN, anorexia nervosa.

Note: "Final inclusion of SNPs" refers to the number of instrumental SNPs retained after instrument selection, linkage disequilibrium clumping, harmonization, and quality-control procedures for each exposure–outcome pair. All GWAS summary statistics were derived from participants of European ancestry. F value refers to the F-statistic used to assess instrument strength; values > 10 were considered indicative of adequate instrument strength and reduced risk of weak-instrument bias.

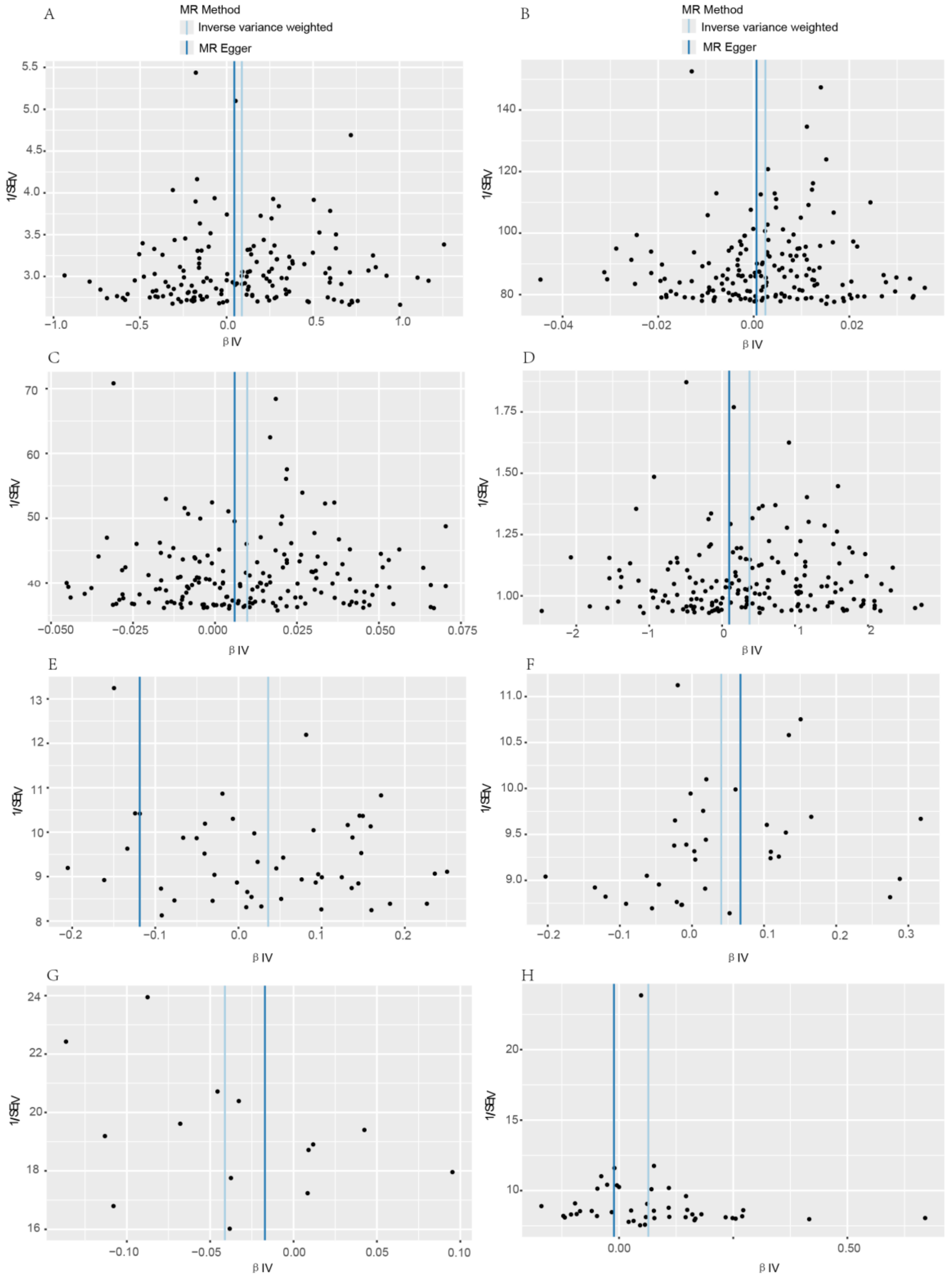

Supplementary figure 1. Funnel plots for selected Mendelian randomization analyses. (A)

MDD-fracture. (B) MDD-osteoporosis. (C) MDD-osteoarthritis. (D) MDD-osteomyelitis. (E) ADHD-fracture. (F) ASD-fracture. (G) AN-fracture. (H) Fracture-MDD. Abbreviations: MDD, major depressive disorder; ADHD, attention-deficit/hyperactivity disorder; ASD, autism spectrum disorder; AN, anorexia nervosa.

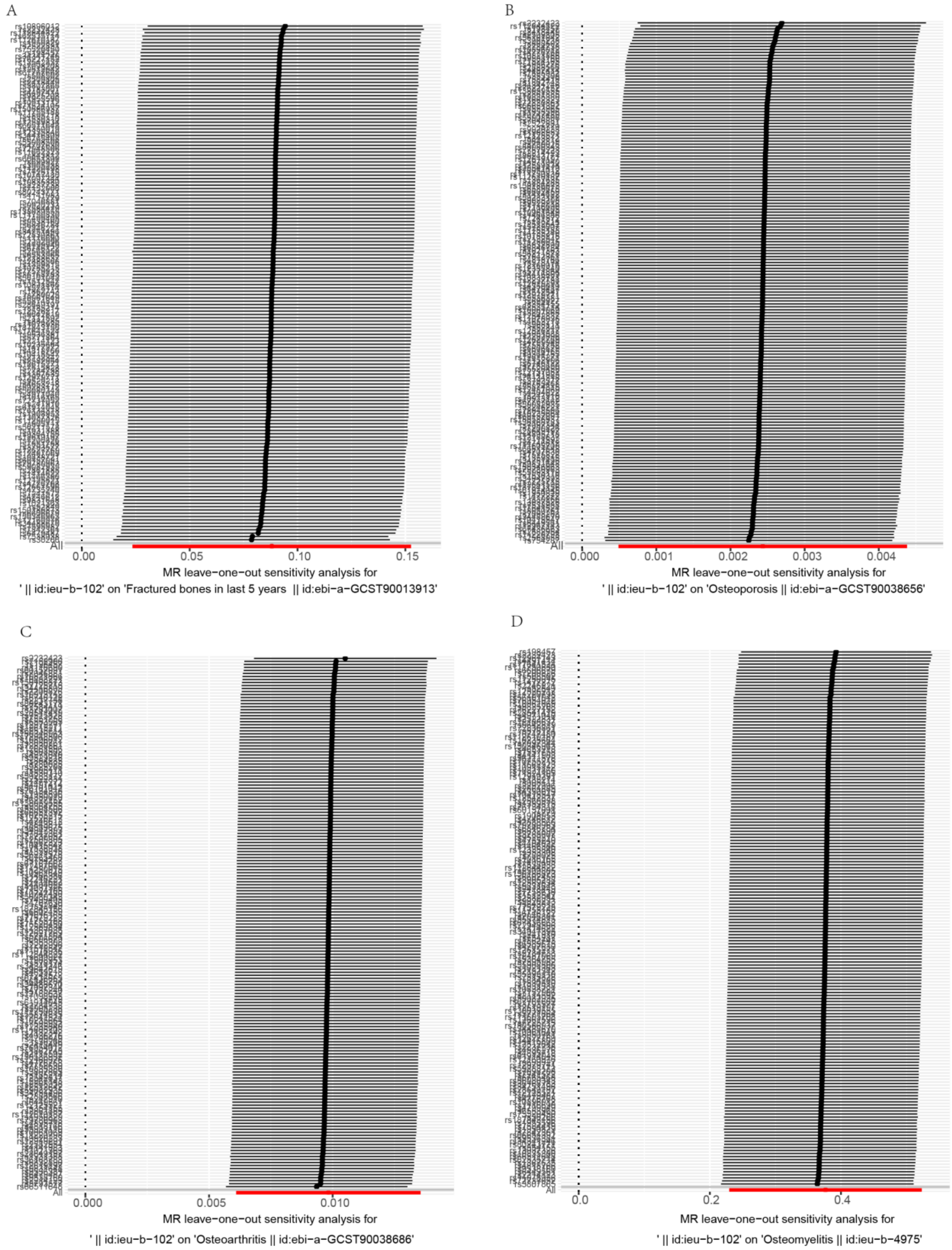

Supplementary figure 2. Sensitivity analysis of the leave one method used in MR analysis. (A) MDD and fractures. (B) MDD and osteoporosis. (C) MDD and Osteoarthritis. (D) MDD and osteomyelitis.

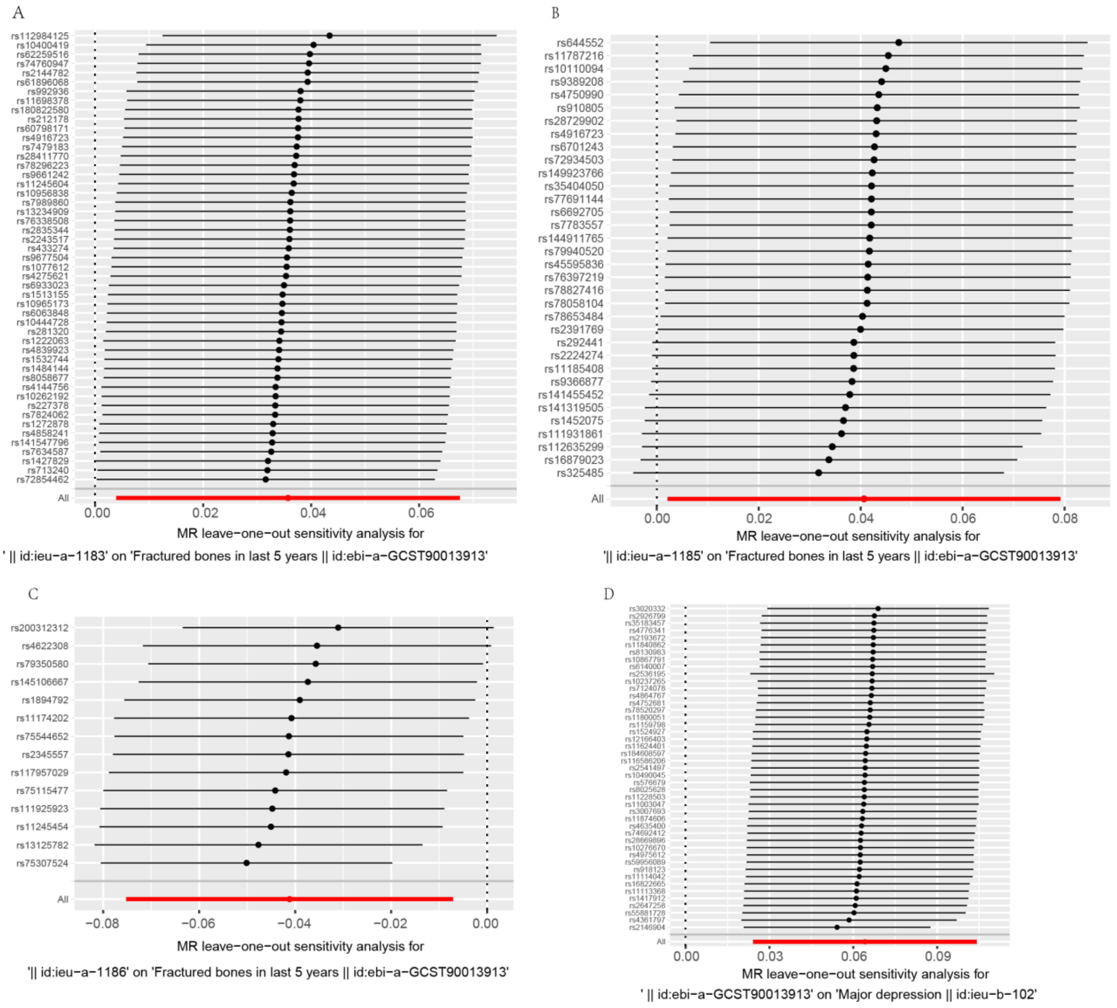

Supplementary figure 3. Sensitivity analysis of the leave one method used in MR analysis. (A) ADHD and fractures. (B) ASD and fractures. (C) AN and fracture. (D) Fracture and MDD.
